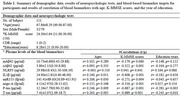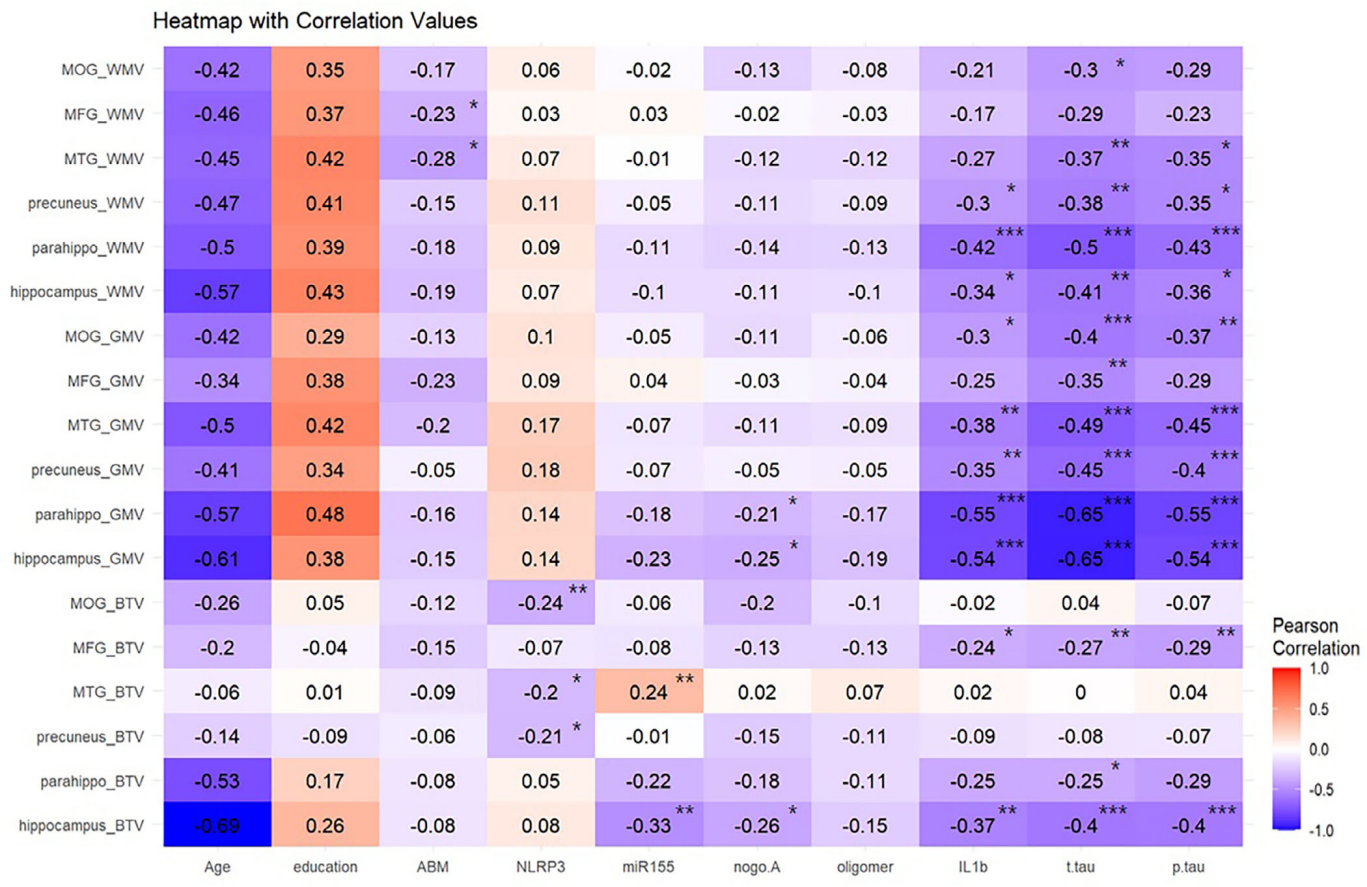# Association between brain tissue volume loss and blood inflammatory biomarkers in cognitively impaired participants with old age

**DOI:** 10.1002/alz70856_100769

**Published:** 2025-12-24

**Authors:** Geon‐Ho Jahng, Yunan Tian, Hak Young Rhee

**Affiliations:** ^1^ Kyung Hee University Hospital at Gangdong, Seoul, Seoul, Korea, Republic of (South); ^2^ Kyung Hee University College of Medicine, Seoul, Seoul, Korea, Republic of (South); ^3^ Kyung Hee University Hospital at Gangdong, Seoul, Korea, Republic of (South)

## Abstract

**Background:**

Blood inflammatory biomarkers have emerged as important tools for the diagnosis, treatment response, and prediction of neurodegenerative diseases. This study evaluated the associations between blood inflammatory biomarkers of interleukin 1‐beta (IL1β), phosphorylated tau (*p*‐tau), total tau (T‐tau), and NACHT, LRR, and PYD domains‐containing protein 3 (NLRP3) and brain tissue volume loss in elderly people.

**Method:**

This study included 111 participants (age, 67.86±8.29 years; 32 males and 79 females). A battery of the following blood inflammatory biomarkers was measured: IL1β, NLRP3, monomer Aβ42 (mAβ), oligomeric Aβ42 (oAβ), miR155, neurite outgrowth inhibitor A (nogo‐A), *p*‐tau, and T‐tau. Three‐dimensional T1‐weight images (3D T1WI) of all participants were prospectively obtained and segmented into gray matter and white matter to measure the gray matter volume (GMV), white matter volume (WMV), the gray‐white matter boundary tissue volume (gwBTV). The association between blood biomarkers and tissue volumes was assessed using voxel‐based and region‐of‐interest analyses.

**Result:**

GMV and BTV significantly decreased as the levels of IL1β and T‐tau increased, while no significant association was found between the level of *p*‐tau and three brain tissue volumes. Three brain tissue volumes were negatively correlated with levels of IL1β, *p*‐tau, and T‐tau in the hippocampus. Levels of IL1β and T‐tau showed a distinct negative association with the three brain tissue volume losses in the hippocampus. In addition, BTV was negatively associated with the level of NLRP3.

**Conclusion:**

The association between brain tissue volume loss and levels of IL1β and T‐tau suggests that IL1β and T‐tau in the blood are potential biomarkers for cognitive impairment in elderly people. Thus, they could be used for future assessment of disease severity and response to treatment after diagnosis in elderly people who might progress to cognitive impairment.